# Differential timing of neurogenesis underlies dorsal-ventral topographic projection of olfactory sensory neurons

**DOI:** 10.1186/s13064-017-0079-0

**Published:** 2017-02-13

**Authors:** Eerdunfu, Naoki Ihara, Bao Ligao, Yuji Ikegaya, Haruki Takeuchi

**Affiliations:** 1 Division of Innate Immunity, Department of Microbiology and Immunology, the Institute of Medical Science, the University of Tokyo, Tokyo, 108-8639 Japan; 2 Laboratory of Chemical Pharmacology, Graduate School of Pharmaceutical Sciences, the University of Tokyo, Tokyo, 113-0033 Japan; 3 Center for Information and Neural Networks, National Institute of Information and Communications Technology, Suita City, Osaka 565-0871 Japan; 4 Japan Science and Technology Agency (JST), PRESTO, 4-1-8 Honcho Kawaguchi, Saitama, 332-0012 Japan

**Keywords:** Olfactory receptor, Olfactory sensory neuron, Neural circuit formation, Topographic map, Zonal organization, Neurogenesis

## Abstract

**Background:**

The mammalian primary olfactory system has a spatially-ordered projection in which olfactory sensory neurons (OSNs) located in the dorsomedial (DM) and ventrolateral (VL) region of the olfactory epithelium (OE) send their axons to the dorsal and ventral region of the olfactory bulb (OB), respectively. We previously found that OSN axonal projections occur sequentially, from the DM to the VL region of the OE. The differential timing of axonal projections is important for olfactory map formation because early-arriving OSN axons secrete guidance cues at the OB to help navigate late-arriving OSN axons. We hypothesized that the differential timing of axonal projections is regulated by the timing of OSN neurogenesis. To test this idea, we investigated spatiotemporal patterns of OSN neurogenesis during olfactory development.

**Methods and results:**

To determine the time of OSN origin, we used two thymidine analogs, BrdU and EdU, which can be incorporated into cells in the S-phase of the cell-cycle. We injected these two analogs at different developmental time points and analyzed distribution patterns of labeled OSNs. We found that OSNs with different dates of origin were differentially distributed in the OE. The majority of OSNs generated at the early stage of development were located in the DM region of the OE, whereas OSNs generated at the later stage of development were preferentially located in the VL region of the OE.

**Conclusions:**

These results indicate that the number of OSNs is sequentially increased from the DM to the VL axis of the OE. Moreover, the temporal sequence of OSN proliferation correlates with that of axonal extension and emergence of glomerular structures in the OB. Thus, we propose that the timing of OSN neurogenesis regulates that of OSN axonal projection and thereby helps preserve the topographic order of the olfactory glomerular map along the dorsal–ventral axis of the OB.

**Electronic supplementary material:**

The online version of this article (doi:10.1186/s13064-017-0079-0) contains supplementary material, which is available to authorized users.

## Background

In the mouse olfactory system, olfactory receptor (OR) genes form a multigene family comprising >1000 genes [1]. Of this rich repertoire of genes, an individual olfactory sensory neuron (OSN) expresses only one functional OR [2]. OSN axons that express a given type of OR converge to a few spatially invariant glomeruli in the olfactory bulb (OB) [3–5], generating an olfactory topographic map. The development of the olfactory topographic map comprises initial global targeting and subsequent activity-dependent refinement [6, 7]. The global targeting process is genetically determined and regulated by two independent mechanisms that control different axes of axonal extension in the OB. Axonal projections along the anterior–posterior axis are dependent on the expressed OR species [8]. Several axon-guidance molecules, such as Neuropilin-1 and Plexin-A1, whose expression levels are regulated by expressed OR molecules have been proposed to participate in anterior–posterior targeting [9–11]. In contrast, as for the dorsal–ventral (D–V) targeting of OSN axons, there is a close correlation between OSN positions in the olfactory epithelium (OE) and glomerular locations in the OB [12]. The OE can be roughly divided into two domains on the basis of genetic markers: dorsal (D)-zone and ventral (V)-zone. D-zone OSNs, which are located in the dorsomedial region of the OE, send their axons to the dorsal region of the OB, whereas V-zone OSNs, which are located in the ventrolateral region of the OE, project their axons to the ventral region of the OB. The expression patterns of OR genes are different between the D- and V-zones. In the D-zone, the expression patterns of OR genes are randomly distributed throughout the OE [13]. However, V-zone specific OR genes show spatially limited expression. Each OR gene possesses a unique expression domain and these domains are arranged in a continuous and overlapping manner within the V-zone [14–16]. Several studies have shown that the topographic order of glomerular locations along the D–V axis of the ventral OB is determined by anatomical locations of OSNs in the V-zone [12, 14].

The topographic projection along the D–V axis of the OB is maintained by axon-axon and axon-target interactions. Several axon guidance molecules have been proposed to be involved in OSN axonal projections along the D–V axis [17–20]. We have previously demonstrated that Neuropilin-2 (Nrp2)/Sema3F repulsive interactions between OSN axons play important roles in preserving the topographic order along the D–V axis of the OB [21]. Nrp2 and Sema3F show complementary, gradient expressions in the OE. More specifically, the expression of Sema3F is high in the D-zone and low in the V-zone, while Nrp2 shows the opposite gradient. We also showed through gain and loss of function experiments that these molecules are necessary for the dorsal–ventral topographic projection in the OB. Further, we found a temporal difference in axonal extensions [21], whereby OSN axons project to the OB from in sequential dorsal to ventral order. These sequential projections are quite important to maintain the topographic order of the olfactory map because early-arriving D-zone OSN axons guide late-arriving V-zone OSN axons by secreting Sema3F. However, it is not clear how the differential timing of these axonal projections is regulated. We hypothesized that the timing of OSN production would be different depending on the location of OSNs in the OE. To test this, we examined spatiotemporal patterns of OSN neurogenesis during olfactory development.

## Materials and methods

### Animals

All experimental procedures were performed with the approval of the Animal Experiment Ethics Committee at the University of Tokyo and according to the University of Tokyo guidelines for the care and use of laboratory animals.

### EdU and BrdU injections

5-Ethynyl-2-deooxyuridine (EdU; Thermo Fisher Scientific) or 5-Bromo-2′-deoxyuridine (BrdU; Sigma-Aldrich) was intraperitoneally injected into mice at embryonic days 11.5, 12.5, 13.5, 15.5, and 17.5, and postnatal days 0 and 2 (50 mg/kg).

### In situ hybridization and immunostaining

In situ hybridization was performed according to previously described methods [21]. Primer sets used to generate RNA probes are listed in Additional file 1: Table S1.

Immunostaining was performed according to previously described methods [22]. Primary antibodies used are as follows: mouse anti-BrdU antibodies (1:500, Sigma-Aldrich); rabbit anti-OMACS antibodies (1:500); goat anti-OCAM antibodies (1:500, R&D systems). Anti-OMACS antibodies were generated by immunizing rabbits with KLH-conjugated synthetic peptides corresponding to 7–29 amino acid residue of the *OMACS* gene (operon biotechnologies). EdU signals were detected with the Click-iT EdU imaging kit (Thermostat Fisher Scientifics). To detect BrdU signal, sections were pre-treated for 1 h in 1.2 N HCl at 37 °C and then rinsed with 0.1 M borate buffer (pH 8.5). After washout with phosphate-buffered saline (PBS), sections were incubated in a detection solution for 30 min. Slides were then washed three times in PBS for 5 min and immunostained with anti-BrdU antibodies.

### Image acquisition and statistical analyses

Optical and fluorescent images were photographed with a BZ-X700 microscope (Keyence). Images of three coronal OE slices were acquired and the number of Br(E)dU-positive OSNs located within the NCAM-positive OE layer was manually counted. To define the OR-zones, serial OE sections at a thickness of 10 μm were used. Each OE section was subjected to in situ hybridization using one of four OR probes (M72, P2, I7 and MOR28). After taking images, expression patterns of the OR genes were compared to determine the boundaries between the OR-zones. Percentages of labeled OSNs within the OR-zones were calculated as the number of labeled OSNs within each OR-zone divided by that in the entire OE region. All statistical analyses were performed with Origin Software (OriginLab).

## Results

**Fig. 1:**
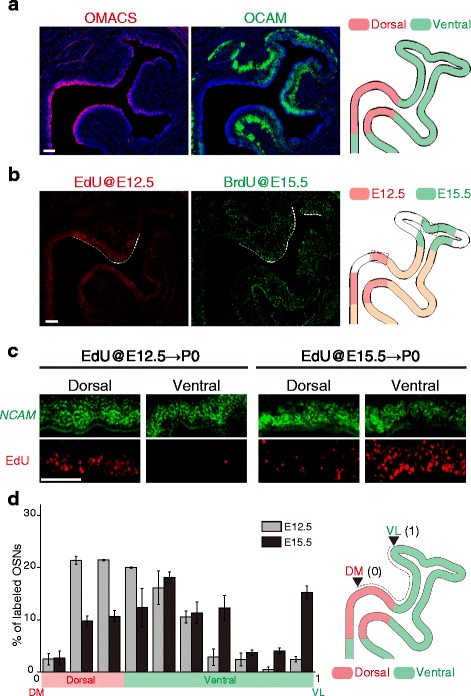
Differential distribution patterns of E12.5- and E15.5-generated OSNs in the OE. **a** A coronal OE section from a postnatal day 0 (P0) mouse, immunostained with antibodies against OMACS (*red*) and OCAM (*green*). The OE section was counterstained with DAPI (*blue*). OMACS and OCAM are markers for the D- and V-zones, respectively. Schematic diagram of an OE section (shown on the *right*). **b** Distribution patterns of E12.5- and E15.5-generated OSNs in the OE. EdU and BrdU were injected at E12.5 and E15.5, respectively. OE sections from P0 mice were subjected to immunostaining with anti-BrdU antibodies and the Click reaction to detect EdU signals. **c** Enlarged images of the D- and V-zones of the OE. *NCAM*, an OSN marker, was detected by fluorescent in situ hybridization. **d** Percentages of OSNs generated at E12.5 and E15.5 were quantified in the OE. The OE sections were divided into ten subregions along with D-V axis. Percentages of labeled OSNs within the subregions were calculated as the number of labeled OSNs within each subregion divided by that in the entire OE region. Schematic diagram of the OE is shown on the *right. n* = 3 slices from 2 to 3 animals, *error bars* indicate SEM. *Scale bars*, 100 μm

The D- and V-zones of the OE could be clearly separated on the basis of the expression of olfactory-specific medium chain acyl-CoA synthetase (OMACS) and olfactory-specific cell adhesion molecule (OCAM) [23–25], as OMACS is expressed specifically in the D-zone and OCAM in the V-zone (Fig. 1a and Additional file 2: Figure S1). Double staining of an OE section with antibodies against OMACS and OCAM delineated clear borders between the D- and V-zones of the OE (Fig. 1a and Additional file 2: Figure S1). To determine the timing of OSN production, we used either BrdU or EdU, which are incorporated into cells during the S-phase of the cell-cycle. EdU and BrdU were injected into pregnant mice at embryonic day 12.5 (E12.5) and E15.5, respectively. Then, distribution patterns of Br(E)dU-positive cells in the OE were analyzed at postnatal day 0 (P0). Using this approach, we observed that Br(E)dU-positive OSNs were not uniformly distributed across the OE. EdU, which was used to label OSNs generated at E12.5, was predominantly detected in the D-zone of the OE, and only lightly visible in the V-zone of the OE. On the other hand, a large number of E15.5-generated OSNs were located in the V-zone of the OE, while only a few were visible in the D-zone of the OE (Fig. 1b, c and d). The differential distribution patterns of Br(E)dU-labeled OSNs suggest that the dynamics of OSN proliferation change during the course of development.

**Fig. 2:**
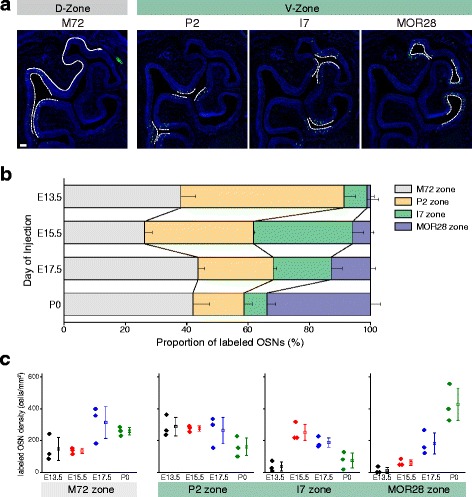
Spatial- and temporal-specific patterns of OSN neurogenesis in the OE. **a** In situ hybridization of OE sections from a P7 mouse using probes for OR genes (M72, P2, I7, and MOR28). The OE was subdivided into four zones based on OR gene expression patterns. The zones defined by the ORs are indicated by *dashed lines*. Note that the M72-area corresponds to the D-zone of the OE. **b** Proportions of EdU-labeled OSNs within the OR-defined zones. EdU injection was performed at E13.5, 15.5, 17.5, and P0, and distribution patterns were analyzed at P7. **c** Quantification of proliferation levels, defined as the density of EdU-labeled OSNs within OR-defined zones. *n* = 3 sections from 3 animals, *error bars* indicate SEM. *Scale bar*, 100 μm

Encouraged by this result, we further examined the relationship between the timing of neurogenesis and OSN localization within the OE. We injected EdU at various developmental time points (E11.5, E13.5, E15.5, E17.5, and P0) and compared distribution patterns of EdU-labeled OSNs in the OE. The zone index [14] is typically used to quantify the location of OSNs. However, we were unable to apply the zone index to our study as it can only be used on OE sections from 3-week-old mice. Thus, we used another metric to quantify OSN location. We subdivided the OE region into four zones based on the expression patterns of the following four well-characterized OR genes: M72, P2, I7, and MOR28. Each of the selected genes showed unique and non-overlapping expression patterns in the OE at P7 (Fig. 2a). We then calculated the proportion of EdU-labeled cells in each zone. As a result, we found that the percentage of E13.5-generated OSNs was much higher in the P2-zone than in the MOR28-zone. In contrast, the percentage of P0-generated OSNs was higher in the MOR28-zone than in the P2-zone (Fig. 2b and c). Since the P2-zone occupies the more dorsomedial region of the OE than the MOR28-zone, these results suggest that OSNs are gradually produced from the dorsomedial (DM) to the ventrolateral (VL) direction within the ventral OE. However, no positional differences were observed in the distribution patterns of EdU-positive OSNs within the M72-zone, which corresponds to the D-zone. In the D-zone, the largest number of EdU-positive OSNs was produced around E17.5 (Fig. 2c). It should be mentioned that no obvious EdU-positive OSNs were visible in E11.5 EdU-injected mice (data not shown). It could be that the incorporation of EdU by OSN precursor cells was diluted due to cell division, to the degree that the EdU signal was reduced to background levels.

**Fig. 3:**
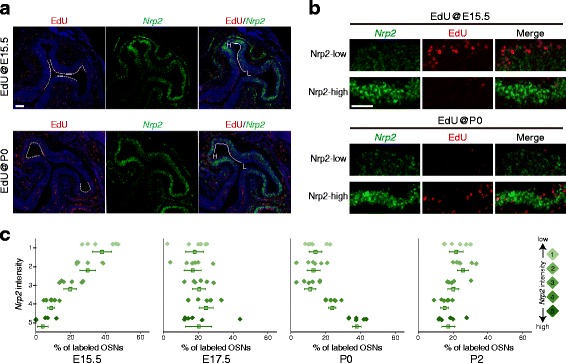
Sequential proliferation of OSNs from DM (Nrp2-low) to VL (Nrp2-high) within the V-zone of the OE. **a** Distribution patterns of E15.5- and P0-generated OSNs in the OE. EdU injection was performed at E15.5 and P0. After the injections, coronal OE sections from P7 mice were subjected to in situ hybridization using probes for *Nrp2*. EdU signals were detected with the Click reaction. EdU-positive OSNs were located in the area indicated by the *dashed lines. Nrp2* can be visualized as being expressed in a DM-low to VL-high gradient. *H* Nrp2-high, *L* Nrp2-low. **b** Enlarged images of Nrp2-high (VL) and Nrp2-low (DL) zones. **c** The kinetics of OSN proliferation were spatially different within the V-zone of the OE. The ventral OE was divided into five subzones according to *Nrp2* expression levels (*right*). The level of proliferation in each subzone was quantified as the percentage of EdU-positive cells. *n* = 6 areas from 3 animals, *error bars* indicate SEM. *Scale bars*, 100 μm

Nrp2 expression can be used as another index of OSN location, as Nrp2 shows a VL-high to DM-low expression gradient in the OE (Fig. 3a). Further, the expression level of Nrp2 determines glomerular locations along the D–V axis of the OB [21]. We then compared distribution patterns of EdU-labeled OSNs and Nrp2 expression (Fig. 3a and b). Using this approach, it was evident that a greater number of E15.5-generated OSNs were located in the Nrp2-low area than in the Nrp2-high area. However, the percentage of P0-generated OSNs was much higher in the Nrp2-high area than in the Nrp2-low area. The percentage of E17.5-generated OSNs was comparable between the two regions (Fig. 3b and c). Together, these results indicate that the area with the most EdU-labeled OSNs shifted from DM to VL within the V-zone of the OE during embryonic development. It should be noted that P2-generated OSNs were uniformly distributed (Fig. 3c). Given that Nrp2-low OSN axons projected to the OB earlier than Nrp2-high OSN axons, it is possible that the timing of OSN axonal projection is determined by that of OSN neurogenesis.

## Discussion

**Fig. 4:**
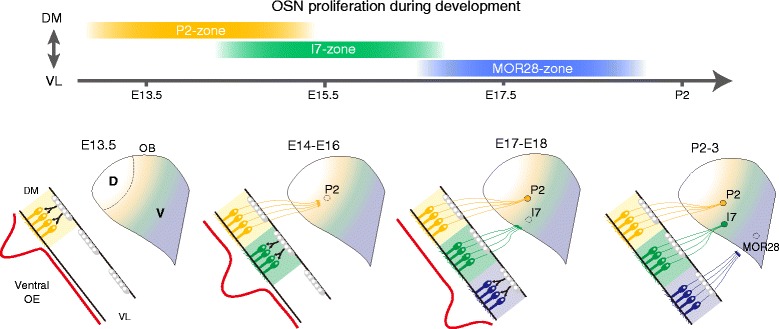
Model depicting development of the olfactory glomerular map along the D-V axis of the OB. At early stages of development, precursor cells within the DM region of the V-zone undergo high rates of cell proliferation, which increases the number of immature OSNs. A wave of cell proliferation travels along the D–V axis of the OE (*top*), from the DM- to VL-region. In accordance with the cell proliferation process, axon extension and glomerular formation occur sequentially, from the dorsal to ventral regions of the OB. The differential timing of OSN neurogenesis may define the timing of glomerular formation, thus contributing to the establishment of a precise olfactory glomerular map along the D–V axis (*bottom*)

The development of neural circuits relies on the timely production of neurons. In this study, we examined spatiotemporal patterns of OSN neurogenesis during olfactory development. In the mouse olfactory system, the positional information of OSNs in the OE regulates the expression of OR genes and axon guidance molecules, thereby correlating OR identity with glomerular locations along the D–V axis. In this study, we provide the first evidence that OSN positional information also controls the timing of OSN proliferation. Support of this was provided by the observation that OSN production rate was not uniform across the OE. Moreover, we found that the area with the most Br(E)dU-labeled OSNs shifted from DM to VL during the course of development, suggesting that cell proliferation propagates in a DM–VL direction across the OE (Fig. 4).

What is the significance of the differential timing of OSN neurogenesis? The timing of neurogenesis is a determinant for cell-fate specification and precise neural circuitry [26, 27]. For example, it has been reported that the timing of neurogenesis determines the location of secondary olfactory mitral cells as well as their axonal projections [28]. However, in the primary olfactory system, region-specific OR gene expression is already observed at E13.5 [29, 30]. Furthermore, other regional markers, such as Nrp2, OMACS, and OCAM, are expressed as early as E12.5 [21, 24]. These observations indicate that the fate of OSNs is already specified at the early embryonic stage. Thus, it is unlikely that the timing of neurogenesis determines the expression of ORs nor does it determine the projection site of OSN axons.

OSN axons project to the OB in a dorsal to ventral direction. The sequential projections of OSN axons are known to be involved in formation of the olfactory map along the D–V axis [21]. Our present study indicated that the sequence of OSN axonal projections correlated with that of OSN proliferation in the V-zone of the OE. This result prompted us to think that the timing of OSN neurogenesis contributed to olfactory map formation through the regulation of axonal projection timing (Fig. 4). It has been reported that glomerular structures first emerge from the anterodorsal region of the OB [31]. Moreover, several experiments using fluorescent protein-tagged OSN axons have revealed that glomeruli for P2, I7, and MOR28 first emerge around E17.5-18.5, P0-1, and P3-5, respectively (Table 1 and Fig. 4) [5, 32–35]. These glomeruli are aligned in the order of P2, I7, and MOR28 along the D–V axis of the OB. Given that a certain number of OSN axons are required to form a glomerular structure, these observations point toward an intriguing possibility that the spatial- and temporal-specific proliferation of OSNs could regulate the timing of glomerular formation by controlling the number of OSN axons at a particular target.

**Table 1:** The timing of OSN proliferation in the OE and glomerular formation in the OB

OE		OB	
OE region	Timing of most active OSN proliferation	OR type	Timing of glomerular formation
M72-zone (D)	ND	M72	PD2-3 [35]
P2-zone (V)	E13.5	P2	E17.5-18.5 [5, 34]
I7-zone (V)	E15.5	I7	PD0-1 [32]
MOR28-zone (V)	P0	MOR28	PD3-5 [33]

*ND* not determined

In contrast to the V-zone of the OE, we were unable to find a positional difference in the rate of OSN production within the D-zone of the OE (Fig. 2b). It could be that different mechanisms are responsible for mediating OSN neurogenesis in the D-zone of the OE. In this study, we observed a burst of proliferation twice in the D-zone: the first one was at E12.5 and the second one was at E17.5 (Figs. 1d and 2c). D-zone ORs are classified into two phylogenetically different groups; Class I and Class II [36]. These two groups constitute distinct glomerular domains in the dorsal OB; the anterior region comprises Class I-ORs while the posterior region comprises Class II-ORs [13, 37]. We assumed that the first burst of proliferation reflects the proliferation for Class I OR-expressing OSNs, and the second one reflects that for Class II OR-expressing OSNs. Comparing the difference in the timing of neurogenesis between Class I OR- and Class II OR-expressing OSNs may give us some insight into the mechanisms underlying the formation of these domains in the OB.

## Additional files

Additional file 1: Table S1. PCR primer sets used to generate RNA probes for in situ hybridization. (DOCX 13 kb)
Additional file 2: Figure S1. Separation of the D- and V-zones of the OE on the basis of markers. (A) Immunostaining of an OE section with antibodies directed against OMACS and OCAM. OMACS and OCAM are markers for the D- and V-zones, respectively. Enlarged photos are shown on the bottom. White dotted lines show the boundaries of the D- and V-zones. The OE section was counterstained with DAPI. (B) Immunostaining of an OE section with antibodies directed against OMACS and NQO1. Both molecules were co-expressed in the D-zone of the OE. Note that OMCAS was expressed not only in the OE but also in the lamina propria. Enlarged photos are shown on the bottom. White dotted lines show the boundaries of the D- and V-zones. The OE section was counterstained with DAPI. (PDF 2969 kb)

## Abbreviations

OB: Olfactory bulb
OCAM: Olfactory-specific cell adhesion molecule
OE: Olfactory epithelium
OMACS: Olfactory-specific medium acyl-CoA synthetase (OMACS)
OR: Olfactory receptor
OSN: Olfactory sensory neuron
