# Supplementary material for: Differential timing of neurogenesis underlies dorsal-ventral topographic projection of olfactory sensory neurons
Source: Neural Dev. 2017 Feb 13;12:2. doi: 10.1186/s13064-017-0079-0 (PMC5307877; doi:10.1186/s13064-017-0079-0)
Supplement: Additional file 1: Table S1. — PCR primer sets used to generate RNA probes for in situ hybridization. (DOCX 13 kb) [file 13064_2017_79_MOESM1_ESM.docx]

Table S1**.** PCR primer sets used to generate RNA probes for *in situ* hybridization

| gene | Forward primer (5’ to 3’) | Reverse primer (5’ to 3’) |
| --- | --- | --- |
| M72 | ACTAACAGGCCAGAGCTCCA | GCTGTAGATCAGGGG |
| P2 | TCCAGGACAAAACCATCTCC | GTGAGGATGGCAG |
| I7 | ACCCTCCACAAACCCATGTA | GAAGCCCCAGTGACAGAGAG |
| MOR28 | TTTTAAATTGTCCTGACAAACTGG | TCTGATTCTCTCAGTCCCTTCA |
| Nrp2 | CTGGTTAGTAGCCGCTCTGG | TTCCCTATCACTCCCTCGAA |
| NCAM | ATTCTTCCTGTGTCAAGTGG | GTTGGCAGTGGCATTCACG |
