# Supplementary figures and images for: Differential timing of neurogenesis underlies dorsal-ventral topographic projection of olfactory sensory neurons
Source: Neural Dev. 2017 Feb 13;12:2. doi: 10.1186/s13064-017-0079-0 (PMC5307877; doi:10.1186/s13064-017-0079-0)

A OMACS/OCAM

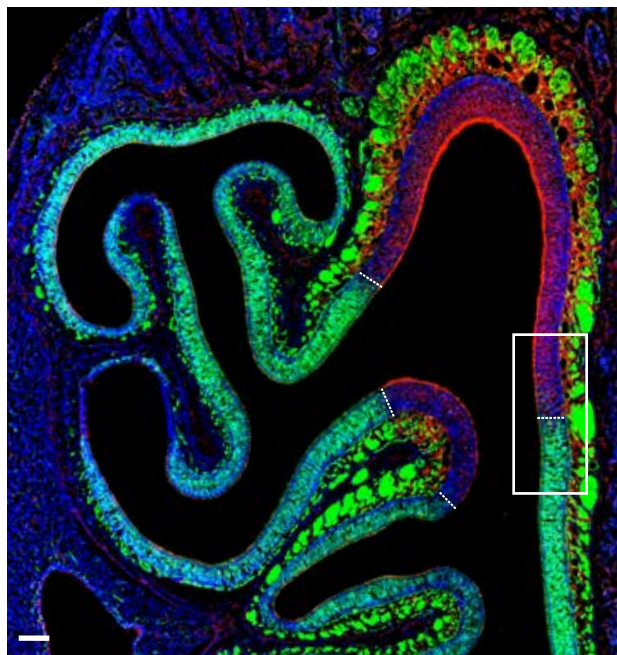

OMACS

OCAM

merge

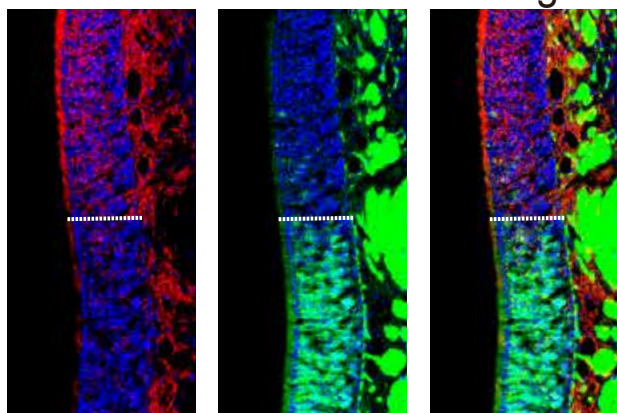

B OMACS/NQO1

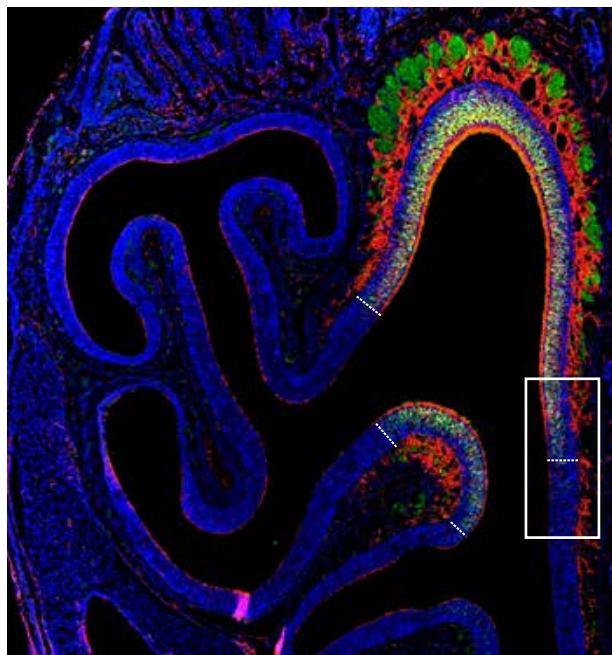

OMACS

NQO1

merge

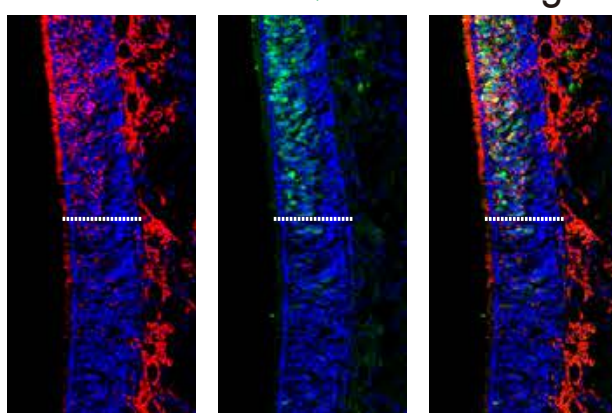

Supplement: Additional file 2: Figure S1. — Separation of the D- and V-zones of the OE on the basis of markers. (A) Immunostaining of an OE section with antibodies directed against OMACS and OCAM. OMACS and OCAM are markers for the D- and V-zones, respectively. Enlarged photos are shown on the bottom. White dotted lines show the boundaries of the D- and V-zones. The OE section was counterstained with DAPI. (B) Immunostaining of an OE section with antibodies directed against OMACS and NQO1. Both molecules were co-expressed in the D-zone of the OE. Note that OMCAS was expressed not only in the OE but also in the lamina propria. Enlarged photos are shown on the bottom. White dotted lines show the boundaries of the D- and V-zones. The OE section was counterstained with DAPI. (PDF 2969 kb) [file 13064_2017_79_MOESM2_ESM.pdf]
